# Carbon nanotubes and graphene towards soft electronics

**DOI:** 10.1186/s40580-014-0015-5

**Published:** 2014-04-25

**Authors:** Sang Hoon Chae, Young Hee Lee

**Affiliations:** 1 Center for Integrated Nanostructure Physics (CINAP), Institute for Basic Science (IBS), Suwon, 440-746 Republic of Korea; 2 Department of Energy Science, Department of Physics, Sungkyunkwan University (SKKU), Suwon, 440-746 Republic of Korea

**Keywords:** Carbon nanotube, Graphene, Nano-carbon, Soft electronics, Flexible, Stretchable, Transparent conducting film, Thin film transistor

## Abstract

Although silicon technology has been the main driving force for miniaturizing device dimensions to improve cost and performance, the current application of Si to soft electronics (flexible and stretchable electronics) is limited due to material rigidity. As a result, various prospective materials have been proposed to overcome the rigidity of conventional Si technology. In particular, nano-carbon materials such as carbon nanotubes (CNTs) and graphene are promising due to outstanding elastic properties as well as an excellent combination of electronic, optoelectronic, and thermal properties compared to conventional rigid silicon. The uniqueness of these nano-carbon materials has opened new possibilities for soft electronics, which is another technological trend in the market. This review covers the recent progress of soft electronics research based on CNTs and graphene. We discuss the strategies for soft electronics with nano-carbon materials and their preparation methods (growth and transfer techniques) to devices as well as the electrical characteristics of transparent conducting films (transparency and sheet resistance) and device performances in field effect transistor (FET) (structure, carrier type, on/off ratio, and mobility). In addition to discussing state of the art performance metrics, we also attempt to clarify trade-off issues and methods to control the trade-off on/off versus mobility). We further demonstrate accomplishments of the CNT network in flexible integrated circuits on plastic substrates that have attractive characteristics. A future research direction is also proposed to overcome current technological obstacles necessary to realize commercially feasible soft electronics.

## 1 Introduction

Since the invention of the transistor, the semiconductor industry has affected nearly every aspect of our daily life [1,2]. One main stream technological trend of the silicon industry is scaling down the device sizes. For instance, the gate length has been reduced down to ~20 nm under current optical lithography technique, and the count of transistors in a commercially available CPU numbers more than 5 billion [3]. In spite of the tremendous progress of miniaturized silicon technology, further development to soft electronics is still limited by the rigidity of the materials themselves. Electronic devices on flexible and stretchable substrates, defined as soft electronics, are contrasted to traditional rigid chips using conventional silicon and metals. The strategies for developing soft electronics are driven by the investigation of new materials which are bendable, twistable, flexible and stretchable. Toward the basic requirement of replacing traditional rigid silicon electronics by new materials, structure engineering, such as structures in “wavy” layouts and the open mesh geometry have also been investigated to achieve stretchability [4–6].

**Figure 1:**
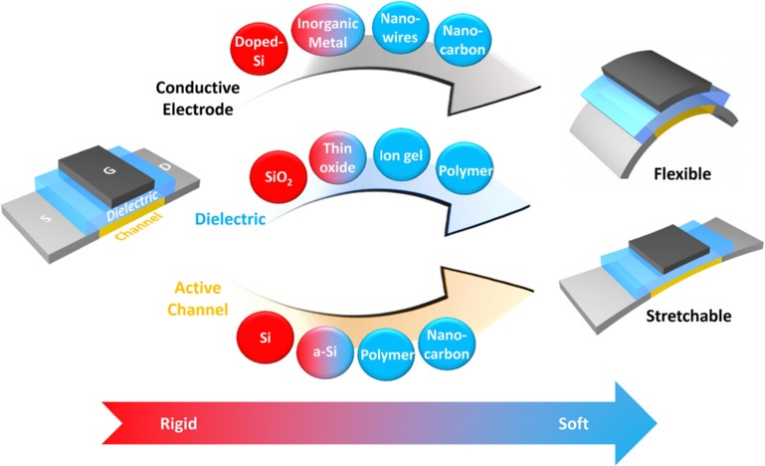
**Classification of materials from rigid to soft.** Conventional Si-based materials need to be replaced by new materials to realize soft (flexible/stretchable) electronics. With good electrical and mechanical properties, materials such as a-Si, organic polymer, nanowires, and nano-carbon materials are good candidates for next-generation soft applications.

Figure 1 shows the development of materials for achieving soft electronics from traditional rigid chips. Amorphous silicon (a-Si), low temperature polycrystalline silicon (p-Si), semiconducting metal oxides, nanowires, and organic semiconductors are promising candidates for flexible electronics from a materials perspective, but several challenges must be overcome prior to their practical use. a-Si is low-cost and is applicable for large-area displays, but suffers from poor mobility and flexibility [7]. Low temperature p-Si has the advantage of relatively high mobility but has low uniformity and processability [8]. Metal oxides are costly due to the shortage of rare earth elements and display poor environmental stability. Polymers have substantial bendability, but have poor mobility and chemical stability.

**Figure 2:**
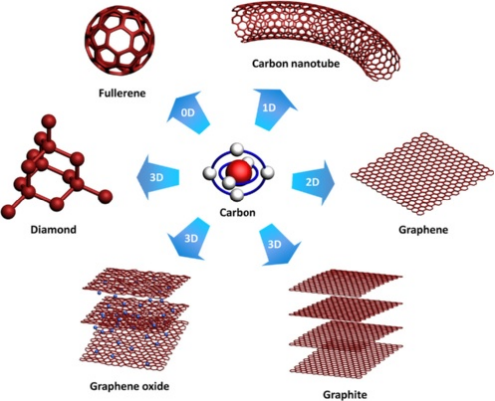
**Carbon-based nanomaterials.** Nano-carbon materials including 0D fullerene, 1D CNT, 2D graphene, 3D graphite, 3D graphene oxide, and 3D diamond are demonstrated.

Nano-carbons such as one-dimensional carbon nanotubes (CNTs) and two-dimensional graphene layers have been widely studied to open a new technology platform based on flexible electronics requiring high transmittance, bendability, and high mobility [9–12]. Figure 2 shows various types of carbon-based materials - fullerene, CNT, graphene, graphite, graphene oxide (GO), and diamond.

The extraordinary electrical, physical, and chemical properties of CNTs and graphene have been attractive since their discoveries. Both materials exhibit outstanding carrier mobility, which is attractive for applications to electronic devices. The carrier mobility in semiconducting single-walled carbon nanotubes (SWCNTs) has been shown to be as high as ~80,000 cm^2^ V^−1^ s^−1^ [13], while the mobility of exfoliated graphene ranges from ~100,000 cm^2^ V^−1^ s^−1^ [14] on insulating substrates to 230,000 cm^2^ V^−1^ s^−1^ in suspended structures [15]. These ultra-high mobility values suggest that these materials have the potential to outperform established materials for next-generation high-speed electronics. The electric current capacity for both CNTs and graphene are reported above 10^9^ A cm^−2^ [16,17]. At room temperature, CNTs exhibit a thermal conductivity up to 3,500 W m^−1^ K^−1^ [18], and graphene has a value of 5,300 W m^−1^ K^−1^ [19] with a high transmittance of nearly 97% [20]. In addition to high flexibility and stretchability, both materials also have superb mechanical strength (Young's modulus of 1.0 TPa and tensile strength of 130 GPa) [21]. For these reasons, CNTs and graphene are regarded as the most promising materials to realize next-generation electronics.

The purpose of this article is to summarize the recent progresses of both CNTs and graphene in soft electronics, and furthermore, to provide guidance for future nano-carbon research by clarifying feasible approaches which will most likely lead to soft applications. We first discuss several successful attempts to synthesize CNTs and graphene. Variations in transfer techniques for both materials are discussed thoroughly. For the use of CNTs and graphene for transparent conducting films (TCFs), the characteristics of TCFs using both nano-carbon materials are compared in depth, together with ITO. Furthermore, various types of field-effect devices using different forms of CNT FETs such as single CNT FET, random network CNT FET, aligned CNT FET, and different forms of graphene FETs such as single layer graphene (SLG), bilayer graphene (BLG), and graphene nanoribbon (GNR) are compared. Moreover, the specific FET device performances related to material preparation and fabrication techniques are also discussed. Finally, the logic level, flexibility, and stretchability of devices with a combination of graphene and CNTs along with their utilizations in logic circuits are further discussed. The systematic deep analyses of the device properties of graphene and CNTs highlight excellent opportunities for future flexible electronics. We conclude with a brief perspective on the research directions of soft electronics in future.

## 2 Review

### 2.1 Material preparations

The preparation techniques for CNTs and graphene are the most important fundamental research areas providing realistic applications. From the discovery of CNTs and graphene, diverse work has been done to improve the quality of the materials (crystallinity and uniformity) and to control other parameters (chirality, density, and doping levels) and morphology (length, area, dimension, and thickness). This section describes some of the most successful methods for synthesis of nano-carbon materials.

#### 2.1.1 Carbon nanotubes

**Figure 3:**
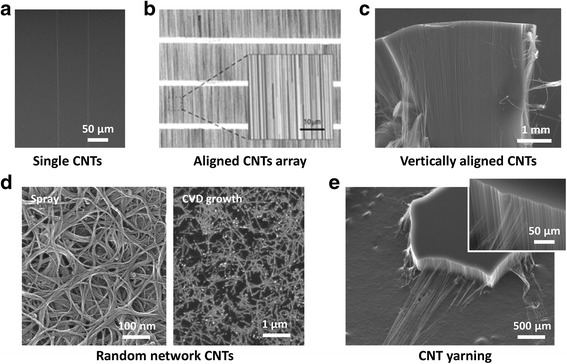
**Various methods of CNT film preparation. a**, CVD-grown aligned individual SWCNTs on SiO_2_ substrate using stable and laminar gas flow. **b**, Aligned array of CNTs on ST-cut quartz with narrow strip pattern of Fe catalyst. Reproduced with permission [32]. Copyright 2007, Nature Publishing Group. **c**, Array of vertically aligned MWCNTs on Fe/Al/SiO_2_ substrate. **d**, Random network SWCNTs prepared by spray of CNT solution (left) and CVD-grown on SiO_2_ substrate (right). **e**, Yarning of vertically aligned MWCNT film.

The CNT synthesis techniques aim to provide control over the tube density, spatial distribution, length, and orientation. Controlling the tube diameter and ratio of semiconducting to metallic SWCNTs have been a critical issue for electrical applications [22,23]. The conventional growth methods for large-scale CNTs include arc discharge, laser ablation, and chemical vapor deposition (CVD) [24–28]. While CNTs grown by arc discharge and laser ablation usually have fewer structural defects than those produced by CVD techniques, the CVD method is intrinsically scalable for realistic applications due to its low setup cost, high production yield, and ease of scale-up. Moreover, long average tube lengths can be obtained from CVD method, which lead to generally better electrical properties in CNTs. The challenge to control alignment and geometry of SWCNTs is mitigated by the CVD method as well. As a one-dimensional material, the as-grown CNTs have various geometries, as shown in Figure 3.

Individual CNTs are horizontally grown on the substrate by CVD, as shown in Figure 3a. Horizontally aligned SWCNTs can be grown using stable and laminar gas flow, which can be determined by the Reynolds number, which depends on volumetric flow rate, viscosity of gases, and the hydraulic diameter of the quartz tube [29,30]. Both the buoyancy effect induced by gas temperature and gas flow stability play a dominant role in preparing batch-scale SWCNT arrays [31]. In Figure 3b shows scanning electron microscopy (SEM) images of an aligned SWCNT film grown from Fe catalyst patterned into narrow stripes oriented perpendicular to the growth direction on quartz [32]. The CVD process on ST-cut quartz wafers using patterned stripes of Fe catalyst leads to the highest levels of alignment and density of CNTs. Linear alignment of individual SWCNTs was achieved with an average diameter of ~1 nm, and a density approaching ~10 SWCNT/μm. Figure 3c shows that vertically stacked CNT films can stand on a SiO_2_ substrate. The CVD growth was carried out on various catalysts, including Fe nano-particles and metal thin films (Fe, Al/Fe, Al_2_O_3_/Co) on Si wafers, quartz, and metal foils to synthesize CNT forest [33,34]. Depending on the collection time, the thickness of CNT films can be changed from micrometers to a few centimeters [35]. Highly-stacked nanotube structures were successfully fabricated on wafer-scale substrates with different thicknesses, which are robust for numerous applications as a conducting film [36,37]. Efficient field emission has been demonstrated where the screening of the field emission current is determined by the ratio of the interlayer spacing to CNT length [38,39]. Figure 3d shows SEM images of random network geometry CNTs. The network geometry can be achieved easily by printing SWCNTs from a solution suspension [40,41]. Solution methods such as spray, filtering, dip-coating, and ink-jet printing have been commonly used for random network type CNT films [42–46]. One serious drawback of the solution approach is the bundling of individual CNTs. This degrades the performance of transparent conducting films (sheet resistance vs transmittance) and transistors (on/off ratio vs mobility) [47]. Random network CNT films prepared directly from CVD or arc discharge can also produce CNT networks and improve the device performance [48,49]. The bundling of CNTs can be avoided and rather clean CNTs can be retained through the CVD method without worrying about the addition of additives that are used in solution approach [41]. By controlling the concentration of catalysts of Fe/Co/Mo, the density of CNTs can be modified, due to increased surface area, pore volume, and catalytic activity [50]. Nevertheless, realizing large-area with good uniformity is still challenging with the CVD method. Owing to their strength, toughness, capabilities of mechanical energy damping, and resistance to knot-induced failure, yarns made from vertically aligned films of MWCNTs are promising multifunctional materials [51–53]. Figure 3e shows an example of the yarning process for a vertically aligned MWCNT film. A beneficial feature of these yarns is the diameter, which can be as little as 2% of the diameter of a human hair, making them ideal as an artificial muscle actuator or artificial muscle, and for storing energy as part of a fiber supercapacitor or battery. MWCNT fibers could also replace rigid metal wires in electronic textiles, such as in heated blankets, where the rigidity of the metal wires can be uncomfortable. Replacing wires with conducting fibers can also provide radio or microwave absorption, electrostatic discharge protection, other types of textile heating, or for simple wiring applications such as headphones where flexibility is important [37,54].

#### 2.1.2 Graphene

Since graphene was first electrically isolated from graphite using a mechanical exfoliation method, many efforts have been studied to synthesize thin graphene films such as the CVD method, reduction of graphene oxide (GO), epitaxial growth on SiC, and chemical molecular assembly method.

**Figure 4:**
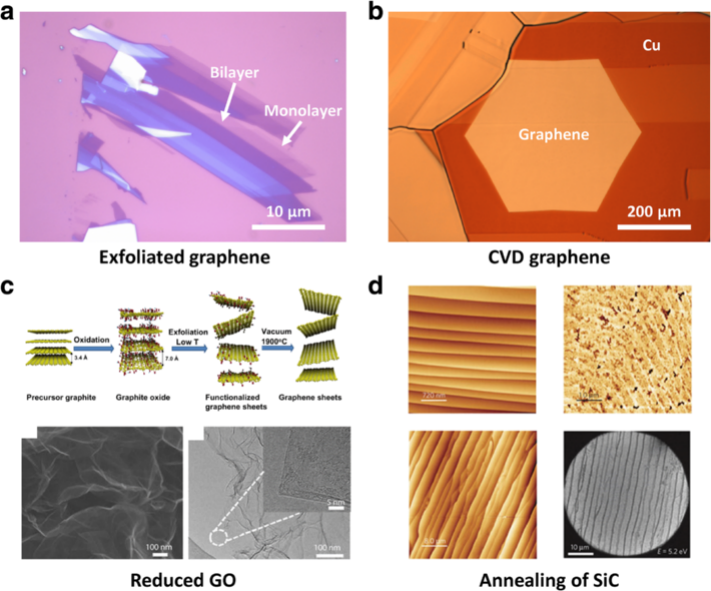
**Various methods of graphene synthesis. a**, Exfoliated graphene (monolayer, bilayer, and other thick layer) obtained by taping from graphite. **b**, Graphene flake is grown on Cu foil by CVD. **c**, Schematic procedure to generate high quality graphene powder obtained from reduced graphite oxide and the electron diffraction pattern. Adapted with permission [73]. **d**, Images of monolayer graphene on 6H–SiC(0001) for explaining epitaxial growth of graphene. Reproduced with permission [74]. Copyright 2009, Nature Publishing Group.

As shown in Figure 4a, the mechanical exfoliation technique offers high quality but small flakes of graphene. Tape was used as the micromechanical cleavage layer to detach graphene samples from graphite. The exfoliation method was followed by the identification and selection of monolayers by using an optical microscopy, scanning electron microscopy (SEM), and atomic force microscopy (AFM) [55,56]. However, the practical use of such a graphene for electronics applications is limited by the tiny size of the exfoliated graphene films, despite their high crystallinity. The preparation of graphene using the CVD method has been reported for the feasible use of graphene [57–59]. Figure 4b shows that the graphene flake was grown on Cu foil under an atmospheric CVD system. The CVD approach is attractive because it allows fabrication over large-area, and expansion of the applicability of graphene to flexible or stretchable devices. Although quality and size of graphene keep improving, field effect mobilities of devices using CVD graphene exhibit still lower values compared to those of devices with exfoliated or epitaxial graphene. Yet, the presence of defects such as point defects, grain boundaries, and wrinkles is unavoidable in the CVD process [60]. Grain boundaries and defects reduce the conductivity of the film and therefore it is highly desired to remove them during growth. Observations and controlling such defects are key research topics in the CVD method. Atomic rearrangement at graphene grain boundaries has been observed using transmission electron microscopy (TEM) and scanning tunneling microscopy (STM). Recent works use optical microscopy to observe the grain boundaries realized by selectively oxidizing the underlying copper foil through graphene grain boundaries functionalized with –O and –OH radicals generated by ultraviolet irradiation [61] and sodium chloride solution [62]. Graphene can be also prepared by a liquid-phase exfoliation or reduction of GO, which has advantages in quantity, yield and cost [63–67]. Large quantities of GO can be prepared by the traditional Brodie and Hummer method, although these methods can be slightly modified to improve the quality of GO [68–71]. Several reducing agents have been used to achieve reduced GO [72]. Although these methods are advantageous for mass production, the complete removal of epoxy and hydroxyl groups and defect generation are an unsolved problem at the present time, unlike the high quality pristine graphene. A simple thermal exfoliation followed by high temperature annealing up to 1500°C in vacuum provides a route of obtaining better quality graphene powder (Figure 4c) [73]. This graphene powder method is challenging but certainly advantageous for conducting film and electrode applications. The fabrication of graphene using the epitaxial growth of graphene directly on rigid insulating silicon carbide (SiC) wafers has been also reported (Figure 4d) [74]. A carbon-included material like SiC is used as a substrate for graphene preparation with high temperature annealing (around 1,500°C) [75]. Graphene obtained with epitaxial growth is highly crystalline, thus is intensely studied to fabricate transistors that operate at high frequencies [76,77]. Wafer-scale graphene can be produced by epitaxial growth on SiC, but those graphenes are not suitable for practical purposes because it is hard to detach graphene from the SiC substrate. Although a solid source molecular beam epitaxy method was also reported to fabricate graphene directly on Si(111), the high cost of molecular beam epitaxy will likely prevent the method from being commercially viable [78].

#### 2.1.3 Transfer methods

Most CVD approaches for synthesizing CNTs and graphene require high temperatures which prevent direct growth of nano-carbon materials on plastic and other soft target substrates. CNTs and graphene located on a catalytic substrate need to be transferred onto a target substrate. Transferring graphene from the metal substrates onto desired substrates without degrading the quality of graphene is the critical step to use CVD-grown graphene for most practical applications.

**Figure 5:**
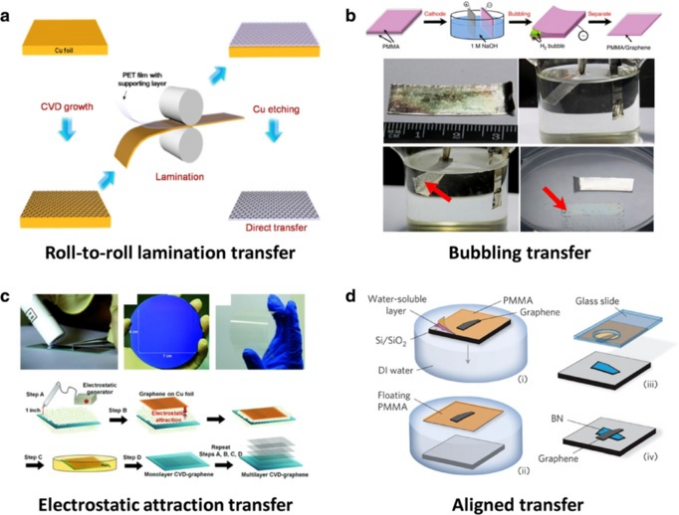
**Various transfer methods of graphene. a**, Schematic demonstration of Roll-to-Roll lamination transfer using a thermal release layer. Adapted with permission [82]. **b**, Schematic and photography images of bubbling process. The PMMA/graphene/Pt was dipped into NaOH solution with a constant current supply. Reproduced with permission [84]. Copyright 2012, Nature Publishing Group. **c**, “Clean-lifting transfer (CLT)” method, which uses electrostatic forces to transfer graphene. Adapted with permission [85]. **d**, Aligned transfer for placing graphene and BN to a desired location. Reproduced with permission [86]. Copyright 2010, Nature Publishing Group.

Wet etching processes are commonly used to detach as-grown materials from the mother substrates using chemical solutions. FeCl_3_ or (NH_4_)_2_S_2_O_8_ are often used for removing Cu, and NaOH or KOH for sapphire [79,80]. The most popular binder to hold graphene during wet etching is poly(methyl methacrylate) (PMMA), but this process unavoidably damages and contaminates the graphene layer with residuals, and is not desirable for scale-up fabrication. The dry printing (or stamping) technique uses polydimethylsiloxane (PDMS) stamp to transfer SWCNTs and graphene films from the growth substrates such as SiO_2_/Si and metal films, still has problems with mechanical damage [81]. The roll to roll (R2R) lamination process can produce a large-area graphene film on flexible substrates [82,83]. The R2R transfer technique uses a thermal release layer as a temporary support and enables the continuous production of graphene film on 44 inch-scale flexible substrates. The synthesized graphene with Cu foil was laminated with the assistance of an adhesive layer, poly(ethylene co-vinyl acetate, EVA) with vinyl acetate (VA) as a supporting layer, to plastic film, followed by Cu etching, as shown in Figure 5a [82]. The transferred graphene film has appropriate uniformity with a resistance deviation of less than 10%. However, the graphene surface is still contaminated by organic adhesive from the thermal release tape using this transfer approach, which may fairly degrade the electrical properties of the film. Undesired mechanical defects also can be caused by this R2R transfer on graphene film. A bubbling method for transferring graphene films to target substrates is nondestructive not only to graphene but also to the mother-substrate (Figure 5b) [84]. The PMMA/graphene/Pt(or Cu, Ni) was dipped into NaOH solution and was used as the cathode with a constant current supply. At the negatively charged cathode, H_2_ gas is produced by a water reduction reaction, and the PMMA/graphene layer detaches from Pt substrate due to the H_2_ bubbles at the interface between the PMMA/graphene and Pt substrate. Damage of the mother-substrate is reduced considerably, and the substrate can be used repeatedly for the next CVD growth. In addition, the transferred graphene is free of metal particles, which are commonly found in graphene transferred by the metal etching process. Figure 5c explains the “clean-lifting transfer (CLT)” method, which uses electrostatic forces to transfer graphene onto target substrates, and doesn’t use a PMMA adhesive layer [85]. An electrostatic generator (SIMCO, 18 kV) was placed at a distance of one inch away from the substrate, then the discharge process occurred via the electrostatic generator, followed by a pressing process to enable more uniform attachment between graphene and substrate. After the Cu foil was etched, the remaining graphene film on the target substrate was rinsed with deionized water to remove the residual etchant. The methods described so far are a rather simple transfer process that does not take account of positioning. There is an interesting transfer method for aligning 2D flakes to a desired location. In order to fabricate stacked graphene on BN devices, a few-micro-size flakes of graphene and BN should be positioned at a desired location (Figure 5d) [86]. Graphene was exfoliated separately onto a polymer stack consisting of a water-soluble polyvinyl alcohol (PVA) and a PMMA layer. When dipped into water, PVA was dissolved and the graphene/PMMA layer was detached from substrate and was floated on the surface of water bath. The PMMA membrane was securely adhered to a holder, which has a tiny hole to identify the top flake onto the PMMA layer during the aligned transfer process. The holder was clamped on the arm of a micro-positioner and then mounted on an optical microscope. The graphene was precisely aligned to the target BN flake by using the microscope to locate the position and the two (PMMA/graphene and BN) brought into contact. The demand for stacked layered structures has been growing [87–90]. A better strategy for transfer in a large-area without damages and residues on graphene is required for profound study.

### 2.2 Carbon-based elements

Common electronic devices require conducting, semiconducting, and insulating materials. For conducting elements, several conducting polymers such as polyacetylene, polypyrrole, polythiophene, polyaniline, and poly(3,4-ethylenedioxythiophene) poly(styrenesulfonate) (PEDOT:PSS) have been investigated for future applications to replace conventional rigid conducting and semiconducting materials [91,92]. However, these polymers have a relatively low electrical conductance and poor stability, compared with metal electrodes [93]. a-Si, p-Si, semiconducting metal oxides, nanowires, and organic semiconductors are promising candidates for the active channel, but several challenges - including rigidity and electrical performance issues - must be overcome prior to practical uses. CNTs and graphene electrodes can be an alternative not only to conducting electrodes but also to a semiconducting channel.

#### 2.2.1 Conducting electrodes

Electrical conducting materials would have potential for consumer applications, such as soft displays, energy generators, and human bio-devices. In such applications, metal oxides such as IZO and ITO are the most widely used materials [94–96]. However, they have several limitations: i) They are costly and a predicted shortage of indium is a concern, and ii) fracture strain less than 1% limits the mechanical ability of flexible devices. Nano-carbon materials can overcome many of these limitations and open a new technology platform due to their outstanding electronic, optoelectronic, thermal, and mechanical properties. Here, we describe nano-carbon materials as conductive electrodes and the development of TCF using CNTs and graphene, where the aim is to replace ITO for certain applications.

**Figure 6:**
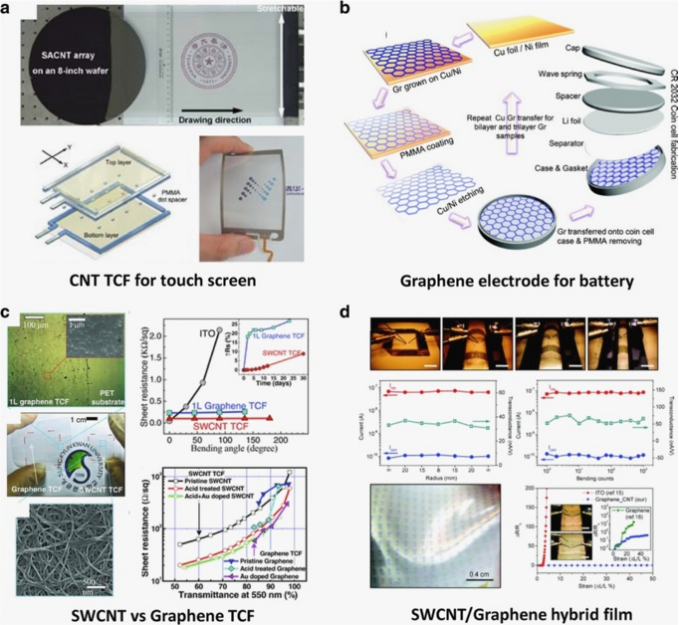
**CNTs and graphene as conducting electrodes. a**, Touch screen using yarned CNT film from vertically aligned CNTs. Adapted with permission [103]. **b**, Li-ion battery using CVD graphene as an electrode. Reproduced with permission [111]. Copyright 2012, American Chemical Society. **c**, Comparison of the properties (bending angle vs sheet resistance, and transmittance vs sheet resistance) of CNT- and graphene-based TCF with ITO film. Adapted with permission [47]. **d**, Mechanical advantage of SWCNT/graphene hybrid electrode. Reproduced with permission [123]. Copyright 2011, American Chemical Society.

During the past few years, much effort has been given in synthesizing CNT films as a conducting element [44,97–99]. Such CNT films have many applications including flexible and stretchable transparent loudspeakers [100], electrodes for LEDs, [101] lithium-ion batteries [102], and touch panels [103]. Figure 6a shows a practical touch panel assembled by directly yarning vertically aligned CNTs. Although the idea of utilizing CNT films as conducting materials is simple, controlling density, average tube length, tube diameter and mixture of metallic and semiconducting CNTs is still challenging. Even with optimized growth conditions, one serious drawback is the relatively high sheet resistance compared to that of conventional ITO [104]. Highly flexible, transparent, and conducting SWCNT films are one of the recent emerging technologies [105–107]. The pristine SWCNT TCF have a reported 360 Ω/sq sheet resistance at transmittance of 90% [43]. This sheet resistance could be dramatically improved by chemical doping treatments. Once such method using nitric acid removes the remaining surfactant from the CNT network and can lower the sheet resistance to a 150 Ω/sq at transmittance of 90% [108]. Further doping with Au^3+^ ions has also been shown to reduce sheet resistance to 110 Ω/sq at a transmittance of 90% [109,110]. While not surpassing the electrical performance of ITO, these films have the advantage of better mechanical stability and are fabricated from a more ubiquitous chemical element, carbon. Figure 6b shows an example that graphene can be used as electrodes to study Li ion diffusion through graphite in lithium-ion batteries [111]. Together with CNTs, graphene is attractive as a conducting film [112,113], due to a large theoretically-predicted conductivity and good chemical stability. In particular, a scalable CVD process to produce large sheets of graphene with high transmittance and robust adhesion to plastic polymers opens the possibility of using graphene in numerous applications in soft electronics. Still the improvement of sheet resistance of the film is an important issue for conducting films. Similar to CNT films, the chemical doping approach has been widely studied for conductivity improvement in graphene films [114–116]. A new approach of layer-by-layer (LbL) doping to improve the conductivity of transparent graphene films has been proposed [117]. Each layer was transferred to a polyethylene terephthalate (PET) substrate followed by AuCl_3_ doping. This approach demonstrates not only improvement of sheet resistance and uniformity but also better environmental stability compared to topmost layer doping. The optimized LbL-doped four-layer graphene shows a sheet resistance of 54 Ω/sq and a transmittance of 85% (at 550 nm) with a robust bending stability. The performance of the graphene conducting films need to be further tuned and improved to meet different requirements of practical flexible products [118,119]. Both CNTs and graphene TCFs have a remarkable spectral response in the UV region, compared to the poor response of ITO films, as shown in Figure 6c [47]. While ITO shows a rapid increase in the sheet resistance due to cracking of the film as the bending angle increases, SWCNTs and graphene films show almost no significant change in the sheet resistance. One drawback of the CNT TCF film is that the performance strongly relies on the dispersion of CNTs in solution. In graphene case, the bottleneck process is the transfer process, which often involves wrinkles and crack formation. Compared to a two-dimensional graphene film, the SWCNT/graphene hybrid electrode is interesting due to its enhanced mechanical properties [120–122]. The SWCNT/graphene hybrid electrode showed a 36% resistance change at a 50% strain, as shown in Figure 6d [123]. The resistance change is remarkably smaller than found in ITO electrodes (i.e., 2000% at 5% strain) and even in a few layers of graphene (i.e., 200% at 30% strain). This superb stretching performance results from the use of graphene and SWCNT network. A continuous and robust contact can be formed between the SWCNT network and the graphene electrode even with graphene layer cracks under strain. This one- and two-dimensional material combination could well provide CNTs and graphene as an appropriate soft and transparent electrode. Table 1 summarizes the transmittance and sheet resistance of various films. It seems that doping is very necessary to reduce sheet resistance. It is also noted that the CNT/graphene hybrid may improve the sheet resistance. This will be a future research direction.

**Table 1:** **Performance comparisons for TCFs based on graphene and carbon nanotubes**

**Material**	**Preparation method**	**Transmittance (% at 300 Ω/sq sheet resistance)**	**Sheet resistance (Ω/sq at 90% transmittance)**	**Flexibility**	**Stretchability**
Random network CNTs [108]	Spray & AuCl_3_ doping	95.7	110	O	O
Yarning CNTs [103]	Laser trimming & Metal deposition	91	208	O	O
CVD Graphene [117]	Layer-by-layer doping	97	108	O	O
CNT-Graphene hybrid [123]	Solid-phase layer-stacking	70	735	O	O
Metal-Graphene hybrid [124]	Metal grid & Graphene transfer	-	20	O	O
ITO [104]	Sputtering	91	80	Poor	-

#### 2.2.2 Active channel – CNT and graphene FETs

Miniaturization is the most important issue not only to increase device integration density but also to improve FET performance for complicated operations. Semiconducting Si technology has given great contributions to society, but now faces scaling which involves heat and power consumption issues due to the fundamental limitations of Si. Atomic-thick nano-carbon materials might satisfy the scaling issue and give great benefits with combination of electrical/mechanical/optical advantages. As an active channel component, SWCNTs and graphene have been studied for fabricating FETs and p − n junctions to demonstrate their potential to outperform established materials for next-generation electronics [125–128]. Here, we discuss extensively the advantages and challenges of such nano-carbon materials for the use of FETs and furthermore their adaptability to silicon technology.

**Figure 7:**
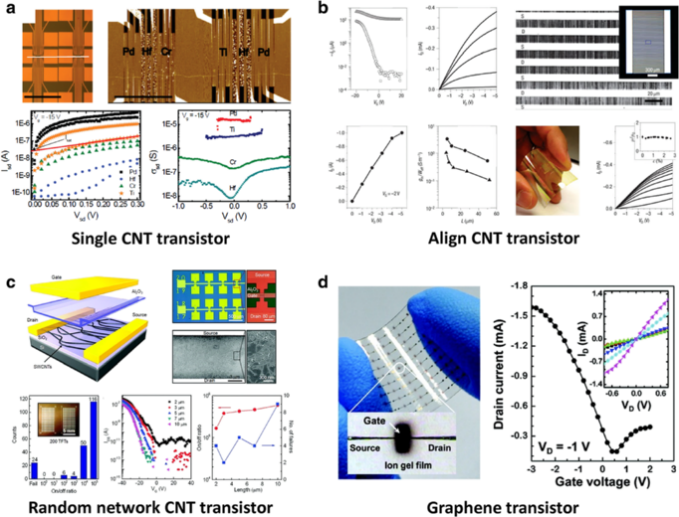
**Morphologies and characteristics of CNTs and graphene FETs. a**, Single CNT transistors with different metal electrodes (Pd, Hf, Cr, and Ti). Reproduced with permission [135]. Copyright 2011, American Chemical Society. **b**, Electrical performance, SEM images, and optical microscopy images of flexible TFTs using aligned CNTs array. Reproduced with permission [32]. Copyright 2007, Nature Publishing Group. **c**, Array of FETs with random network SWCNTs. Reproduced with permission [138]. Copyright 2009, American Chemical Society. **d**, Flexible graphene transistor with ion gel dielectric. Reproduced with permission [140]. Copyright 2010, American Chemical Society.

Figure 7 shows that various kinds of FETs using nano-carbon materials-based active channel. Diverse geometries of FETs based on semiconducting SWCNTs have been the subject of intensive research [129–131]. An individual SWCNT FET shows favorable device characteristics such as large on-off ratio (>10^5^), at room-temperature operation [132–134]. With single CNT studies, it has also been demonstrated that the saturated on-current level can be simply determined from the work function difference between the CNT and metal or Schottky barrier height formed at the junction, as shown in Figure 7a [135]. For fabricating this transistor, e-beam lithography is used to pattern the electrodes to desired positions, but has limitations for realistic multi-array transistors. An alternative easy fabrication method without e-beam lithography is required for large-scale integration for practical electronic device applications. Although isolated SWCNTs are not relevant to future applications at their current stage, numerous works show that the aligned arrays of SWCNTs or random networks can serve as an active channel component. Figure 7b shows FETs with aligned arrays of SWCNTs. The use of dense aligned arrays of linear SWCNTs was used as an effective semiconducting channel suitable for integration into transistors and other classes of electronic devices [32]. The tubes were parallel to one another to better than 0.1 degree. The average CNT density can be as high as 10 SWCNT/μm, and the film provides good device-level performance characteristics with mobility of ~1,000 cm^2^ V^−1^ s^−1^ [136,137]. Figure 7c shows an array of FETs with random network SWCNTs that were synthesized on a catalyst (0.01 M of ferrocene) array by using a plasma-enhanced chemical vapor deposition (PECVD) method at low temperature (450°C) [138]. SWCNTs network was placed between the source and drain electrodes and played a role of active channel path. This random network type morphology has the potential applicability from CNT thin film transistors (TFTs) to large-scale flexible electronics due to its good uniformity and processability over a large-area, which is alternative to conventional organic or other classes of semiconductors for integrated circuitry applications [126,139]. However, the gate modulation is degraded due to the inclusion of some metallic CNTs in the channel. Strategies to reduce metallic CNTs in the channel will be discussed in the next Section 3.2.1. Figure 7d shows an example of graphene channel FETs on a flexible plastic substrate [140]. In graphene, the charge carriers in the two-dimensional (2D) channel can change from electrons to holes subject to electrostatic gate with a minimum density (or Dirac) point characterizing the transition [127,141–143]. The experimental graphene FETs have extremely large mobility compared to SWCNT FETs, while on/off ratio is as low as ~10 due to zero band gap. Despite low on/off ratio, high transconductances and current saturation are achieved, making graphene devices suited for analogue applications [144].

##### 2.2.2.1 Performance control – on/off ratio control

One of the key issues in high-performance TFTs is high on/off ratio for efficient switching behavior. In the case of a CNT channel, the as-grown CNT network usually contains both semiconducting and metallic CNTs [145]. These metallic CNT paths reduce the on/off ratio of the transistor [146]. Since controlling the ratio of semiconducting to metallic CNTs leads to a trade-off between on/off ratio and charge carrier mobility of a transistor, engineering the proper parameter is important in terms of the type of applications. In the case of zero band gap graphene, opening the band gap is a big challenge in the way of achieving a higher on/off ratio in transistors [127]. Here, we introduce several strategies for increasing the on/off ratio of a transistor. In CNTs, electrical thinning and selective channel cutting, and separation approaches are described below. BLG and nanoribbon approaches will be discussed for increasing the on/off ratio in graphene transistors.

**Figure 8:**
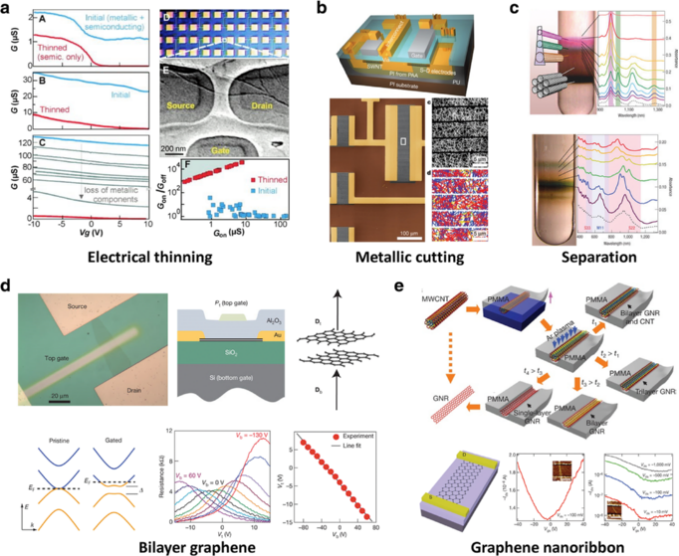
**Various methods of improving on/off ratio of FET based on CNTs and graphene. a**, Thinning of MWCNTs and CNT bundles by applying bias. Reproduced with permission [147]. Copyright 2001, American Association for the Advancement of Science. **b**, Schematic and SEM image of a region of the random network SWCNT channel. A striping technique was used to cut metallic CNT paths. Reproduced with permission [149]. Copyright 2008, Nature Publishing Group. **c**, Separation of semiconducting CNTs and metallic CNTs by density-gradient method. Reproduced with permission [153]. Copyright 2006, Nature Publishing Group. **d**, BLG transistor with top and bottom gate to open band gap. Applying perpendicular field from bottom gate, band gap of the BLG can opened up to 250 meV. Reproduced with permission [157]. Copyright 2009, Nature Publishing Group. **e**, Graphene nanoribbons with a width below 10 nm were obtained by upzipping CNTs. By narrowing the width of graphene to a few nanometers, a quantum confinement effect of carriers happens to open the band gap. Reproduced with permission [162]. Copyright 2009, Nature Publishing Group.

One method to obtain high on/off ratio involves electrical thinning of the thick MWCNTs and CNT bundles, as shown in Figure 8a [147]. The electrical thinning process involves sweeping the drain voltage from 0 V to negative values while holding the gate voltage at a just above the threshold. Multiple sweeps with increasing voltage eventually eliminate metallic CNT channels or thin nanotubes (or bundles) to increase on/off ratio [32,148]. After this procedure the off-state current in the devices is reduced to values consistent with semiconducting CNTs alone. A striping technique was used to cut metallic CNT paths [123,149]. Figure 8b shows the schematic image and SEM image of a region of the random network SWCNT channel. By inserting the cutting line perpendicular to the channel length direction, the metallic CNTs can be terminated and the on/off ratio increases. The critically important role of the cutting width in determining the electrical characteristics can be quantified. For cutting widths of 5 mm, the etched lines increase the on/off ratio by up to four orders of magnitude, while reducing the transconductance by only 40%. It is now possible to obtain uniform CNT thin films with only semiconducting behavior by the techniques of semiconducting/metallic CNT separation in solution [150–152]. The purification processes produce separated CNTs in solution of the same chirality, diameter, length and semiconducting/metallic type. A self-sorting method to achieve a chirality separated CNT thin film by controlling surface chemistry and a further large-scale demonstration was reported in Figure 8c [153]. The representative techniques are density gradient ultracentrifugation (DGU) and gel chromatography, which can produce >99% semiconducting CNTs and continue to improve. Despite the quite low productivity, yield, and high process cost, this DGU technique appears to be the most promising method to prepare semiconducting CNT materials [153]. The gel chromatography separation method, much simpler than DGU method, is based on the strength of the structure-dependent interaction of CNTs with an allyl dextran-based gel [152]. TFTs based on such separated CNTs also provide high on/off ratio. BLG has a unique dispersion relationship whereby application of a strong transverse electric field breaks electron–hole inversion symmetry [154–156]. Experimentally, it has been reported that an optical bandgap of ∼ 250 meV is possible. The effective electrical gap is smaller than the reported optical gap, typically due to the presence of disorder and sample imhomogeneities. Even so, large improvements in on/off ratios and the existence of an insulating state at charge neutrality have been observed (Figure 8d) [157]. In these dual-gate BLG transistors, on/off ratios of ∼ 100 and ~2000 at room temperature and 20 K have been reported, respectively [158]. BLG is disadvantageous compared to graphene monolayer since acoustic-phonon scattering is increased strongly, optical-phonon scattering is reduced, and a parabolic band dispersion near the band edge reduces carrier mobility compared with monolayer graphene [159]. Moreover, the band structure of BLG can be modified, with a larger bandgap possible by applying a combination of strain (along z axis) and an electrical field. However, this approach is unfeasible with current technology. A new strategy demonstrated that benzyl viologen (BV) as an electron-donating group and bis(trifluoromethanesulfonyl)imide (TFSI) as an electron-withdrawing group are conjugated on the top and bottom sides of bilayer graphene to open the band gap [160,161]. This compensation doping induces a high local electric field in the bilayer, but has the limitation of weak field-effect due to a large disorder potential. The graphene nanoribbon (GNR) strategy is to ideally introduce a quantum confinement effect of carriers to open the band gap by narrowing the width of graphene to a nanometer scale. In reality, this strategy is limited by fabrication procedures. Instead of confinement-induced gap, this leads to a coulomb blockade effect that is strongly enhanced for dimensions below 20 nm. Graphene nanoribbons with a width below 10 nm can be obtained by upzipping CNTs (Figure 8e) [162] and by solution-phase stripping from bulk graphite [163]. The GNR transistors exhibited an on-off ratio of ∼ 10^7^ at room temperature [162–165]. Similar to the GNR method, graphene with a nanomesh structure can open up a band gap and shows an on/off ratio of >10^2^ in a large sheet of graphene [166,167]. However, these GNR and graphene nanomesh transistors have poor on-state conductivity and cannot be used for high-speed devices unless a new method is found due to reduce scattering at the edges. The band gap of graphene can be modulated by chemical and physical doping processes. Band gaps of boron- and nitrogen-doped graphene transistors showed an on/off ratio of >100 [168,169]. It also has been reported that by patterned adsorption of atomic H onto the graphene surface, surface absorption can induce a band gap in graphene of at least 450 meV around the Fermi level [170]. Yet, again the degradation of mobility due to sp^3^ hybridization with atomic H makes this approach impractical.

##### 2.2.2.2 Performance Control – Polarity Control

Although CNTs and graphene intrinsically have an ambipolar transport property, both show p-type behavior under ambient conditions due to contacts, doping by oxidizing acids, or doping by the adsorption of atmospheric oxygen molecules and/or moisture. It is important to control the carrier type of nano-carbon transistors for applying “complementary metal-oxide-semiconductor (CMOS) technology” because high noise immunity and low static power consumption are critical issues in the modern semiconductor industry. Therefore, it is desired to control the major carrier types of CNTs and graphene FETs by chemical and/or nonchemical doping methods. Here, we introduce several polarity control methods to modify the majority carriers in CNT- and graphene-based transistors such as chemical doping, oxygen doping, electrostatic doping, trap charge-induced doping, and metal work function engineering.

**Figure 9:**
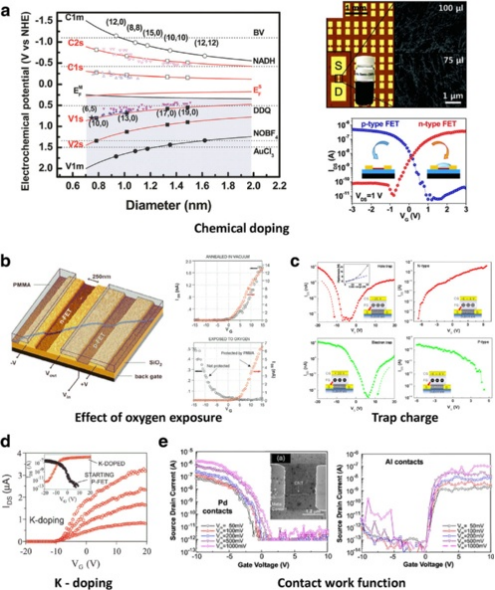
**Various methods of polarity control of FETs based on CNTs and graphene. a**, Redox potential of nanotubes as a function of the diameter (left). This Reproduced with permission [108]. Copyright 2010, Royal Society of Chemistry. Array of n-type CNT transistor by precisely positioning an air-stable BV. Reproduced with permission [181]. Copyright 2011, American Chemical Society. **b**, Effect of oxygen on p-doping. I-V curves of originally p-type CNT FET, with the nanotube capped with PMMA, have been converted to n-type. Reproduced with permission [187]. Copyright 2001, American Chemical Society. **c**, The type conversion of CNT FETs by trap layer-induced electrostatic doping. Adapted with permission [190]. **d**, I-V characteristic of an initially p-type characteristic in SWCNT FET, gradually changed to n-type caused by increasing amounts of K. Reproduced with permission [187]. Copyright 2001, American Chemical Society. **e**, Polarity control by metal (Pd and Al) work function. Reproduced with permission [193]. Copyright 2005, American Institute of Physics.

In order to have n-type conversion and p-type enhancement behavior in CNTs under ambient conditions, various chemical doping strategies have been investigated [171–179]. The choice of chemical dopant is complicated by the fact that the redox potential of CNTs is strongly diameter-dependent, as shown in Figure 9a [108]. The values in parentheses indicate the chiral index of the SWCNTs and the reduction potentials of dopants (BV, NADH, DDQ, NOBF_4_, and AuCl_3_) are also indicated as dotted lines. As shown in Figure 9a, the Au^3+^ ion has the large reduction potential of 1.50 V, which acts as p-type doping in CNTs. BV has an oxidation potential of −1.1 V, which implies that BV can act as an n-type dopants. BV donates electrons to the empty conduction band of semiconducting CNTs [180]. The right panel of Figure 9a shows an example of n-type CNT transistor by precisely positioning BV with inkjet printing on CNTs channel region [181]. Using β-nicotinamide adenine dinucleotide (reduced dipotassium salt, NADH), a type conversion in CNTs is also demonstrated distinctly [182]. A reduction potential of tetrafluorotetracyano-p-quinodimethane (F4TCNQ) in the range of 0.1 V to 0.2 V makes it an electron extractor and p-type dopant [183]. For graphene, it has been demonstrated that the work function of CVD graphene can be modulated up to 1.1 eV with BV doping [184]. Similarly, other work showed GO doping with Au allowed control of the work function [185]. For BLG, surface chemical doping in BLG can be utilized to induce a vertical displacement field. Interestingly, tunable Dirac points can be rationally controlled by the amount of BV doping, providing complementary inverter circuits [186]. Figure 9b shows a simple way to control polarity by just annealing the p-CNT FET in vacuum, converting it to an n-CNT FET [187]. One of the reasons for having p-character in CNT FETs is due to the interaction with O_2_ physisorbs on the CNT surface [148,188,189]. Originally a p-type CNT FET was converted to n-type after annealing process for removing O_2_ molecules [187]. It has been shown that the type conversion of CNT FETs could be possible by electrostatic doping using a charge-trap layer between the gate electrode and CNT channel [190,191]. Figure 9c shows the transfer characteristics of p-type and n-type CNT FETs converted using an Au floating gate. At high negative gate bias range, positive charges are trapped in the trap layer, and the threshold voltage is shifted in the negative bias direction. Therefore, the FETs show n-type characteristics in relatively small gate voltage sweep range. On the contrary, when high positive gate bias is initially applied, which traps the negative charges, the FETs show p-type characteristics in a relatively small gate voltage sweep range. Figure 9d shows the electrical performance of an initially p-type characteristic as it is gradually changed to n-type via increasing amounts of K on the nanotube [187,192]. Potassium ions have a high oxidation potential of −0.7 V and act as an electron donor (n-type dopants) for CNTs. Logic circuits and pn junctions were fabricated by covering half of a CNT FET with PMMA and K-doping the exposed regions. The electrical polarity of SWCNT FETs can be affected by the work function of the contact metal, especially by the contact barrier control for the injection of carriers [148,175,193]. Figure 9e shows the transfer characteristics of CNT FETs using different metal contact electrodes such as Pd and Al [193]. The transfer characteristics show the presence of a p-type on-state but no n-branch in the case of high-work function metals such as Pd and Ti, ambipolar behavior in the case of Mg, and n-type only behavior in the case of Ca electrodes [194]. By varying the work function, the band alignment for a Mg-contacted device has efficient hole and electron injection, resulting in ambipolar characteristics. Conversely, due to work function and surface dipole formation, CNTs contacted by Ca electrodes have a suppressed p-type branch due to large energy barrier for holes. Although this method works to control the injection of carriers in single devices, the use of different metal electrodes in high-density devices is commercially unreasonable and resulting devices still have highly variable contact properties.

Numerous efforts have been made to get higher on/off ratios and better control of carrier type in nano-carbon transistors. In order to understand advantages and disadvantages for CNT and graphene FETs, a side-by-side comparison is required. Table 2 shows the comparison for FET performance of CNTs and graphene devices. CNT FET and graphene devices exhibit output performances in a different manner. Moreover, the performances are distinct in different types of FET devices consisting of different forms of CNTs (single CNT, aligned CNT network, random CNT network) and graphene (CVD graphene, exfoliated BLG, GNR) with different gate structures. Nevertheless, a clear trade-off behavior between on/off ratio and mobility for each device was shown.

**Table 2:** **Device performance of various CNTs and graphene FETs**

**Channel**	**Preparation method**	**Transistor structure**	**Gate dielectric**	**Gate length (μm)**	**Carrier type**	**On/Off ratio**	**Mobility (cm** ^**2**^ **/Vs)**
Single CNT [32]	CVD on quartz	Back gate	SiO_2_	5	p-type	10^5^	636^(C)^
Aligned CNTs [32]	Electrical breakdown	Back gate	HfO_2_	12	p-type	2 → 10^4^	570^(C)^ → 200^(C)^
Random network CNTs [149]	Channel cutting	Top gate	HfO_2_	100	p-type	10 → 10^4^	200^(C)^ → 80^(C)^
Random network CNTs [153]	97% separated CNTs	Back gate	SiO_2_	20	p-type	10^4^	20^(p)^
Random network CNTs [181]	Viologen doped CNTs	Back gate	HfO_2_	9	p → n-type	10^3^	2^(p)^
Exfoliated graphene [141]	Monolayer graphene	Back gate	SiO_2_	4	Ambipolar	10	10,000^(p)^
CVD grown graphene [195]	Monolayer graphene	Back gate	SiO_2_	5	Ambipolar	10	1,100^(p)^
Exfoliated graphene [158]	Bilayer graphene	Dual gate	SiO_2_ (Back) HfO_2_ (Top)	1.6	Ambipolar	5 → 100	-
Graphene nanoribbon [162]	16 →6 nm nanoribbon	Back gate	SiO_2_	0.25	Ambipolar → p-type	1.5 → 100	-

p: Parallel plate Model, c: Cylindrical Model, h: Hole Mobility, e: Electron Mobility.

### 2.3 Flexible electronics

#### 2.3.1 Integrated logic circuits

Next-generation military and industrial radio-frequency (RF) surveillance systems will benefit from flexibility and stretchability of circuits for increased resilience. A realistic short- and medium-term goal for carbon electronics is utilizing the combination of electrical, mechanical, and optical properties of CNTs and graphene thin films to replace organic semiconductors and a-Si in these flexible/stretchable systems [196–204]. In this section, we introduce recent progress for integrating high-quality circuits on plastic substrates.

**Figure 10:**
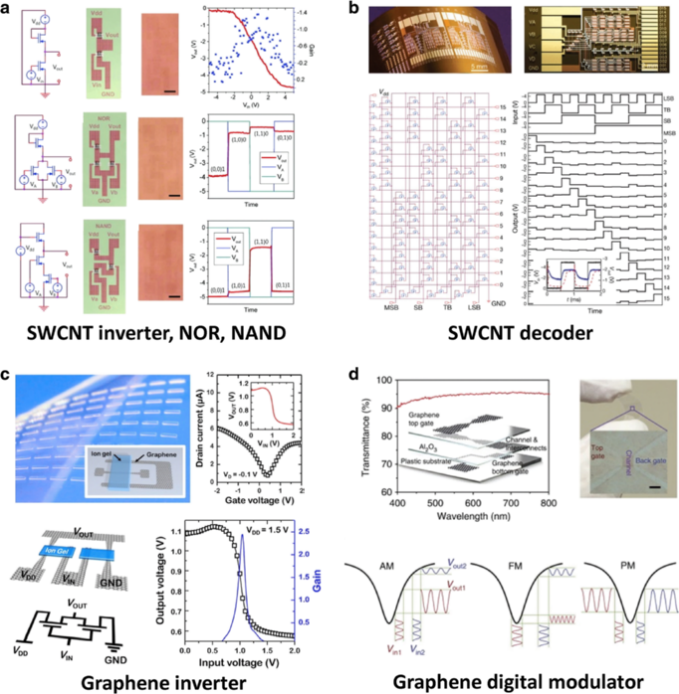
**Flexible logic circuits using CNTs and graphene. a**, Transparent and flexible logic circuits (inverter, NAND, and NOR) using graphene as electrodes and random network CNTs as the channel. Reproduced with permission [123]. Copyright 2011, American Chemical Society. **b**, Flexible four-bit row decoder circuit using SWCNT channel and metal electrodes. Reproduced with permission [149]. Copyright 2008, Nature Publishing Group. **c**, Flexible complementary graphene inverters prepared on plastic substrate with ion-gel gate dielectric. Reproduced with permission [206]. Copyright 2012, American Chemical Society. **d**, Transparent and flexible all-graphene digital modulator for quaternary digital modulations. Reproduced with permission [212]. Copyright 2012, Nature Publishing Group.

Figure 10a shows an integrated circuit fabricated with monolayer graphene as the electrodes and a SWCNT network for the channel [123]. Using this layout, transparent logic circuit arrays (inverters, NOR gates, and NAND gates) using SWCNT-channel/graphene-electrodes transistors were fabricated with a high yield of 80%. The authors connected two p-type transistors to create a PMOS inverter with gain of approximately 1.4, with an operating voltage range of 0–5 V. PMOS NOR and NAND logic gates were similarly constructed using three SWCNT/graphene transistors. The graphene electrode and the SWCNT network channel are desirable not only for flexible and stretchable electronics, but also for use with invisible electronics due to the high transparency of atomically thin materials. Figure 10b shows a flexible four-bit row decoder circuit using SWCNT as the channel and metal electrodes [149]. A binary-encoded input of four data bits is successfully decoded using this decoder circuit. Due to the high mobility of the SWCNT thin films, even with critical dimensions (100 μm) these decoder circuits can successfully operate in the kHz region. With such large channel lengths, cheap and scalable patterning methods such as screen printing are possible. More complex device structures are also easily possible such as master–slave delay flip-flops and 21-stage ring oscillators which were fabricated on PEN substrates [205]. Figure 10c demonstrates flexible complementary graphene inverters prepared on a plastic substrate by connecting two graphene transistors with a coplanar gate configuration. Fabrication was achieved using only two materials: graphene and an ion gel gate dielectric [206]. Unlike conventional solid state dielectrics, the operation of ion-gel gated transistors is based on the formation of a high capacitance electric double layer (EDL) under an electric field. The graphene inverter operates uniquely with two identical ambipolar transistors, unlike complementary inverters based on separate n- and p-channel transistors. Also in contrast to typical CMOS inverters, the output voltage did not saturate to zero or the supply voltage (V_DD_) due to the zero band gap of graphene [206]. With an estimated maximum voltage gain of 2.6, the technology is sufficient to drive subsequent components in logic circuits. Graphene-based frequency doublers and modulators on rigid substrates have been reported to demonstrate the feasible usage of graphene in analogue electronics [207–211]. Figure 10d shows a flexible all-graphene modulator circuit for quaternary digital modulations, which can encode two bits of information per symbol [212]. A couple of transistors are required for these two quaternary modulations.

#### 2.3.2 Other Flexible Applications

Applications ranging from flexible solar cells, displays, e-papers, wearable and biomedical skin-like devices open up new opportunities in the field of electronics. In this section, we describe applications of several flexible devices possible with carbon electronics jsuch as sensors, LEDs, RF devices, stimulators, and memory devices.

**Figure 11:**
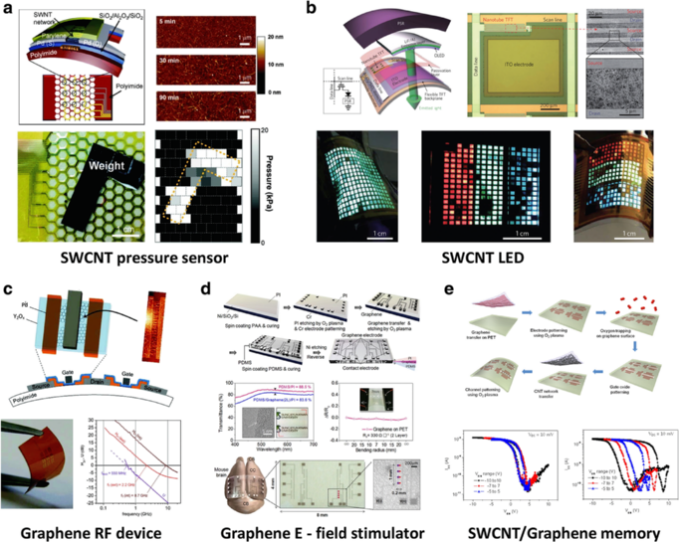
**Various flexible applications using CNTs and graphene. a**, Active-matrix of SWCNT TFTs for a pressure sensor device. Reproduced with permission [213]. Copyright 2011, American Chemical Society. **b**, Flexible active-matrix design using SWCNTs as the channel material of the TFTs in OLEDs. Reproduced with permission [215]. Copyright 2013, Nature Publishing Group. **c**, Flexible RF device using solution-based graphene. Graphene is an ideal material for high-speed communication systems owing to its uniquely high carrier mobility. Reproduced with permission [217]. Copyright 2012, American Chemical Society. **d**, Flexible, transparent, and noncytotoxic graphene electric field stimulator. Reproduced with permission [222]. Copyright 2013, American Chemical Society. **e**, Transparent and flexible memory devices using SWCNT channel and graphene electrodes. The oxygen-decorated graphene electrode revealed an initially large hysteresis in SWCNT/graphene TFT. Adapted with permission [223].

As an example of further applications of flexible devices, Figure 11a demonstrates an active-matrix backplane for an artificial electronic skin (e-skin) device, capable of spatial touch mapping [213]. The SWCNT TFTs are used for a mechanically flexible backplane with polyimide as a support substrate. The polyimide film substrate was utilized as a honeycomb mesh structure to make the substrate more robust against strain. Each pixel of pressure sensor is actively controlled by a SWCNT TFT. The sensor sensitivity shows ∼ 30 μSkPa^−1^, which is three times larger than previous NW-based sensors [214]. Figure 11b shows the flexible active-matrix design with SWCNTs as the channel material. In these devices, high current drive is needed to actively switch OLEDs [215]. Each pixel is controlled by a SWCNT TFT that acts as a switch for an active-matrix of OLED and pressure sensor. Alternating current electroluminescence devices on flexible PET substrates were also demonstrated based on monolayer graphene electrodes [216]. Graphene seems to be an ideal material for high-speed systems owing to its extremely high carrier mobility. Despite poor switching behavior of graphene transistors limits their usage in digital/logic applications, they are still promising in the analogue/RF applications due to their atomic-thick layout that allows for shorter scaling of channel length. The combination of high speed and flexibility is a big challenge for flexible graphene RF devices [217–221]. RF devices using graphene have achieved cut-off frequencies between 100–300 GHz. Figure 11c shows the flexible solution-based graphene transistors at GHz frequencies with a current gain cut-off frequency of 2.2 GHz and a power gain cut-off frequency of 550 MHz [217]. Noninvasive probing and manipulation of biological tissue is another field where graphene is useful. Figure 11d reports a nonvascular surgical method to increase cerebral blood volume using a flexible, transparent, and biocompatible graphene electrical field stimulator [222]. The flexible graphene stimulator was placed onto the cortical brain without tissue damage or unnecessary neuronal activation. A noncontact electric field was applied at a specific local blood vessel to detect effective cerebral blood volume increases in mouse brains using *in vivo* optical recordings of signal imaging. In Figure 11e, transparent and flexible memory devices were fabricated using graphene electrodes and SWCNT channel [223]. The original electrical characteristics of the FET using graphene electrode without ozone treatment show small hysteresis. When the graphene gate was treated under an ozone generator, oxygen atoms and graphene have bonding as C-O-C, C = O, and C-OH, which acted as charge trap sites. The FET with oxygen-decorated graphene electrode exhibits large hysteresis. This hysteresis-controllable FET can act as memory device, and showed no degradation of transmittance after oxygen decoration. This result is noticeable, compared to Au and Al nanoparticle trap layers that provided an 11.4% and 25% decrease in transmittance, respectively [224]. Flexible organic resistive memory devices with multilayer graphene electrodes were also reported [225]. Memory devices using a graphene oxide film were also fabricated on flexible substrates with reliable memory performance in terms of retention and endurance [226].

### 2.4 Stretchable electronics

Stretchability is a key parameter in the development of wearable devices that can be embedded into clothes and garments or even attached directly to the skin, where high levels of strain will be encountered. Possible applications include the human-friendly devices for detecting human motions, monitoring health system, and healing. In addition to flexibility, all these stretchable applications demand tolerance of large levels of strain (> > 1%) without fracture or significant degradation in electronic properties. The mainstream strategy to realize improved stretchability focuses on the development of stretchable materials including organic polymers, networks of 1-D wires, and nano-carbons [227–231]. Owing to the difficulties in developing new stretchable materials, geometrical engineering of the structures also needs to be addressed [6]. For example, ultrathin buckled geometries and pre-strained geometrically wavy materials offer stretchability with applied strain [232–235]. These devices can be integrated into larger systems containing conventional rigid materials. In this section, we introduce developed classes of material-based stretchable devices that use CNTs and graphene thin films on elastomer substrates.

#### 2.4.1 Stretchable conducting films

**Figure 12:**
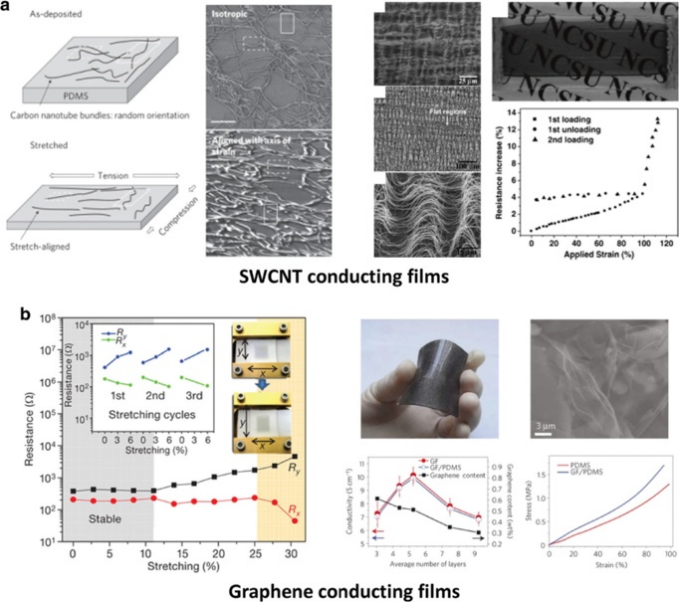
**Stretchable conducting films using CNTs and graphene. a**, Transparent, conducting spray-deposited films of SWCNTs that can be rendered stretchable by applying strain along each axis (left). Reproduced with permission [239]. Copyright 2011, Nature Publishing Group. Wavy ribbons of CNTs are embedded in elastomeric substrates to fabricate stretchable conductors (right). Adapted with permission [231]. **b**, Stretchable conducting films using few-layer CVD grown graphene (left). 3D-graphene macroscopic structure with a foam-like network graphene (right). Reproduced with permission [240,241]. Copyright 2009 and 2011, Nature Publishing Group.

Loading a SWCNT random network onto an elastomeric substrate simply affords a stretchable conducting film with the ability to accommodate strains greater than 20% [236–238]. The left panel of Figure 12a shows transparent, conducting spray-deposited films of SWCNTs that can be stretched by applying strain along each axis [239]. This stretchable SWCNT film accommodates the stretchability by up to 150% with conductivities as high as 2,200 S cm^−1^ at the strain of 150%. This property can be utilized to construct strain sensors, with performance comparable to conventional metal-strain gauges. Using a nonlinear buckling process as shown in the right panel of Figure 12a, ribbon arrays of CNT films can be modified into a “wavy” layout [231]. With a pre-strain (100%) method, the wavy CNT ribbon can accommodate large stretching with the 4.1% resistance increases when the wavy CNT ribbon is stretched to the pre-strain stage. Applied strains lead to a reversible deformation of these buckled patterns which change the electrical properties. Together with the good optical and electrical properties, graphene films have excellent mechanical properties applicable to stretchable electrodes. One such example consists of few-layer CVD grown graphene films transferred onto elastic substrates, as shown in the left panel of Figure 12b [240]. The transferred film on an unstrained substrate recovers its original resistance after stretching by ~6%. In this work, the authors also transferred the film to pre-strained (12%) substrates to enhance the electromechanical stabilities. Both longitudinal and transverse resistances (R_y_ and R_x_) were stable up to ~11% stretching with only one order of magnitude change at ~25% stretching. 3D-graphene macroscopic structures formed with a foam-like network of graphene was also developed using template-directed CVD (right panel of Figure 12b) [241]. The composites fabricated by this approach are a monolithic 3D-graphene network, in which electrical and mechanical properties were improved by using continuous CVD grown graphene building blocks. The results of graphene composites show stretchability over 50% with resistance changes stable after the fifth cycle of stretch-release.

#### 2.4.2 Stretchable applications

Extreme difficulties are associated with the development of complete sets of stretchable electronics because all elements of the system need to be stretched out together. For instance, currently available carbon-based devices such as TFTs usually exhibit limited flexibility and stretchability owing to the use of fragile oxide dielectrics such as Al_2_O_3_ and SiO_2_. Polymer dielectrics have modest electrical performance despite their excellent bendability [242]. In this section, we introduce several strategies to fabricate stretchable devices using CNTs and graphene.

Reproduced with permission [246] Copyright 2011, Nature Publishing Group.

**Figure 13:**
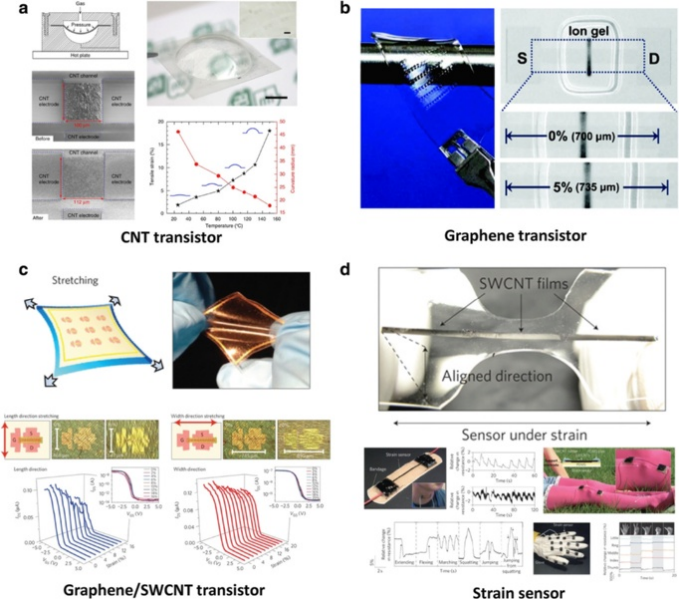
**Stretchable applications using CNTs and graphene. a**, Transparent and soft integrated circuits with random network SWCNT channel and PMMA dielectric layer on the PEN substrate. Reproduced with permission [243]. Copyright 2013, Nature Publishing Group. **b**, Graphene FET array on a stretchable rubber substrate with ion-gel dielectric. Reproduced with permission [244]. Copyright 2011, American Chemical Society. **c**, Stretchable and transparent TFTs combining SWCNTs/graphene with a geometrically wrinkled Al_2_O_3_ dielectric layer. Reproduced with permission [245]. Copyright 2013, Nature Publishing Group. **d**, Wearable and stretchable strain sensors fabricated from thin films of aligned SWCNTs. Reproduced with permission [246]. Copyright 2011, Nature Publishing Group.

**Figure 14:**
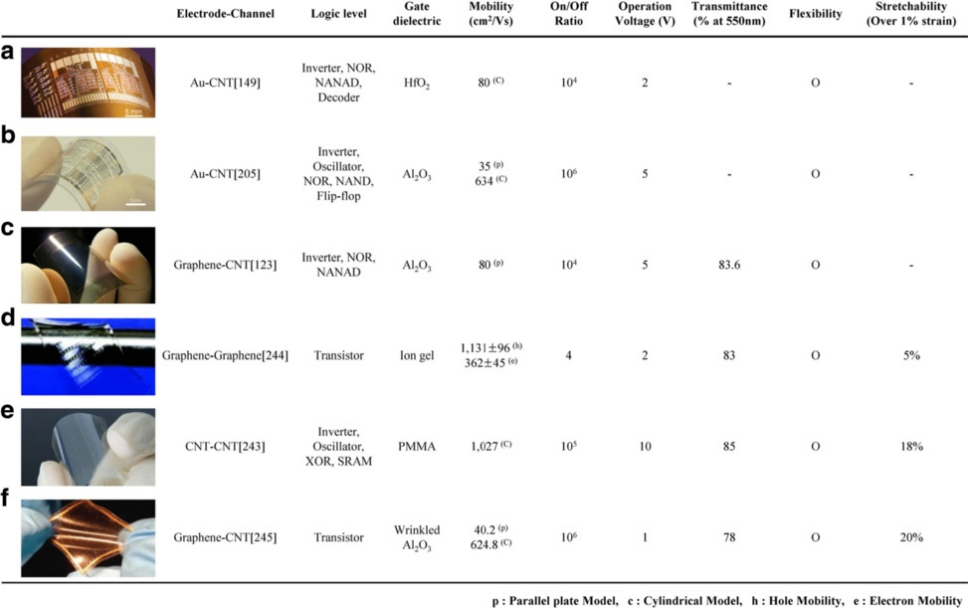
**Performance comparisons between graphene and carbon nanotube logic circuits in terms of their logic level, device characteristics, flexibility, and stretchability. a**, Integrated circuits (inverter, NOR, NAND, and Decoder) with random network SWCNT channel on flexible plastic substrates. Since this device uses metal electrodes, there is no transmittance data. Reproduced with permission [149]. Copyright 2008, Nature Publishing Group. **b**, Flexible integrated circuits (inverter, oscillator, NOR, NAND, and Flip-flop) with random network SWCNT channel on the PEN substrate. Reproduced with permission [205]. Copyright 2011, Nature Publishing Group. **c**, Transparent and flexible logic circuits (inverter, NOR, and NAND) using graphene as electrodes and random network SWCNTs as an active channel. Reproduced with permission [123]. Copyright 2011, American Chemical Society. **d**, Graphene FET array on a stretchable rubber substrate with ion-gel dielectric. Reproduced with permission [244]. Copyright 2011, American Chemical Society. **e**, Transparent and soft integrated circuits (inverter, oscillator, XOR, and SRAM) with random network SWCNT channel and PMMA dielectric layer on the PEN substrate. Reproduced with permission [243]. Copyright 2013, Nature Publishing Group. **f**, Stretchable and transparent TFTs combining SWCNT/graphene with a geometrically wrinkled Al_2_O_3_ dielectric layer. Reproduced with permission [245]. Copyright 2013, Nature Publishing Group.

Figure 13a shows transparent and stretchable integrated circuits composed of CNTs and polymer dielectric [243]. The active channel and electrodes were all fabricated from CNTs (semiconducting and metallic), with PMMA dielectric layer and a plastic substrates. Although these were fabricated on plastic substrate, thermo-pressure was used for forming dorm-shape biaxial strain. The devices exhibit biaxial stretchability of up to 18% and the level of logic circuits include inverters, ring oscillators, NOR, NAND, XOR gates, and static random access memory (SRAM) cells. In Figure 13b, a graphene FET array on a stretchable rubber substrate with ion-gel dielectric is introduced [244]. Such all-graphene devices (graphene composes both the channel and electrodes) exhibit hole and electron mobilities of ~1188 and ~422 cm^2^V^−1^ s^−1^, respectively with stable operation up to 5% stretching. Although the stretchability of transistors is moderate, impressively the electrical properties were invariant even after 1000 cycles. Figure 13c shows a new approach for preparing a wrinkled gate dielectric using a transfer method to maximize the performance of the oxide without compromising the ability to stretch and bend [245]. A 50 nm aluminum oxide (Al_2_O_3_) layer was deposited onto rough Cu foil using atomic layer deposition. After coating with PMMA, Cu foil was chemically etched, and the Al_2_O_3_ layer was then transferred as dielectric layer. This transferred Al_2_O_3_ layer was wrinkled with a “wavy” structure, which was robust under high tensile strain. The resulting TFTs exhibited device-acceptable electrical performance with small gate leakage current due to the build-in air gap between wrinkled Al_2_O_3_ and graphene gate. The devices were stretched along the length direction (16% strain) and along the width direction (20% strain), as shown in Figure 13c. The devices were stretched and released up to a maximum of 1,000 times without deterioration. Figure 13d shows a class of wearable and stretchable devices fabricated from thin films of aligned SWCNTs [246]. When stretched, the films fracture into gaps and islands with tube bundles bridging the gaps. This mechanism allows the films to act as strain sensors with capabilities extending up to 280% strain, which is 50 times more than conventional metal strain gauges, with high durability (10,000 cycles at 150% strain), and fast response (delay time of 14 ms). When the CNT sensors were assembled on stockings, bandages and gloves to fabricate devices, the devices were able to detect human movement, typing, breathing and speech, each unique applications useful for developing human-friendly and bio-integrated devices [239]. Figure 14 shows a summary of the flexible/stretchable device layouts and circuit levels of devices using nano-carbon, followed by the demonstrations of electrical, optical and mechanical properties.

## 3 Conclusions

### 3.1 Summary and prospects

We have reviewed the current status of CNTs and graphene in diverse applications of soft electronics from material preparation to performance in logic circuits. Low-dimensional carbon materials exhibit superb electronic properties and promising performance and are attractive for future electronics. Methods for synthesizing one-dimensional CNT and two-dimensional graphene films, as well as procedures for device fabrication on soft substrates have been discussed here. Both CNTs and graphene exemplify TCF properties including a high operational flexibility and stretchability that are not accessible with transparent ITO electrodes. Likewise, field effect mobilities of carbon-based transistors have reached levels unfeasible by organic semiconductors/a-Si. CNT FETs, whether composed of a single CNT, aligned CNTs, or random network CNTs, show high on/off ratio and mobility. Graphene FETs provide extremely high mobility but poor on/off ratio due to zero band gap. Engineering for on/off ratio increase and carrier polarity control were summarized. For applications in active electronics, SWCNT and graphene transistors can be assembled on a variety of substrates including flexible plastic and stretchable elastomers. Various complex integrated circuits based on nano-carbon materials have been demonstrated in the literature, as well. Each of these topics requires significant future exploration in order to realize commercialized applications of the immense potential of nano-carbon in next-generation electronics.

In spite of recent progress demonstrating the unique advantages of CNTs and graphene, the possible applications, social influence, addressable markets, and related economic issues will eventually decide the success of these nano-carbon materials. Both have unique and superb properties which open the possibility for soft electronics. Nevertheless, applications are limited by a different set of factors. Assemblies of CNTs are practical compared to the use of individual CNTs, but require the positioning of the CNTs in a specific direction, with desired density, and of desired metallicity/chirality. Methods to achieve this control are a current hot topic, but adoption of a particular method will require a high yield for industrial utilization even in niche applications. Conversely, graphene can be prepared in a large-area format. Yet, the transfer to a desired substrate may provoke damage in the graphene layer and degrade device performance. Therefore, developing a smart way of assembling CNTs to maximize the device performance and robust method of transfer of large-area graphene are two key ingredients that are unsolved but required for application. On a systems level, future electronics including biomedical applications with biocompatibility will require further research. For instance, CNTs and graphene combine synergistically, showing better flexibility and stretchability with no degradation of electrical performance when engineered to maximize potential. Additionally, combining both stretchable materials and stretchable geometries can allow for extremely stretchable systems. Aside from the engineering challenges of applying nano-carbon to soft electronics, CNTs and graphene are outstanding materials for demonstrating a number of basic science concepts in the fields of quantum electrodynamics, quantum optics, and quantum chemistry. Controlled synthesis and application of monolayer materials also allows exploration into a new class of vertical tunneling devices. Aside from carbon, other classes of graphene-like 2D materials such as transition-metal dichalcogenide (TMD) materials and boron nitride (BN), might also be promising in the field of soft electronics when a band gap or other electrical/mechanical properties are required. These related engineering opportunities in areas with the broad range of influential research topics provides strong motivation for continued efforts in human-friendly soft electronics.
